# Supplementary material for: The ER membrane protein complex restricts mitophagy by controlling BNIP3 turnover
Source: EMBO J. 2023 Dec 15;43(1):32–60. doi: 10.1038/s44318-023-00006-z (PMC10883272; doi:10.1038/s44318-023-00006-z)

The ER membrane protein complex restricts mitophagy by controlling BNIP3 turnover

Jose M Delgado[1], Logan Wallace Shepard[1], Sarah W Lamson[1], Samantha L Liu[1], Christopher J Shoemaker[1,2]*

* Christopher.J.Shoemaker@Dartmouth.edu (to C.J.S)

TABLE OF CONTENTS

Appendix Figure S1……………………………………………………………………Page 2-3

Appendix Figure S1, related to figure 7.

(A) MDA-MB-231 cells expressing mt-Keima were transduced the indicated sgRNAs. On day 8 post-transduction, cells were incubated in normoxic and hypoxic conditions for 18hr prior to flow cytometry. Bar graphs represent mean +/- SEM from 3 independent experiments. Statistical analysis was performed using two-way ANOVA with Tukey's post-test. ***, $p < 0.001$; *, $p < 0.05$. (n > 10,000 cells)

(B) MDA-MB-231 mt-Keima cells were transduced with the indicated sgRNAs. On day 8 post-transduction, cells were treated with vehicle (DMSO), MLN-4924 (1μM), and CB-5083 (1μM) for 18hr prior to analysis by flow cytometry. (n > 10,000 cells)

(C) MDA-MB-231 cells expressing mt-Keima were transduced the indicated dual sgRNAs. On day 8 post-transduction, cells were treated with Baf-A1 (100nM) or BTZ (100nM) 18hr prior to flow cytometry. Bar graphs represent mean +/- SEM from 3 independent experiments. Statistical analysis was performed using two-way ANOVA with Tukey's post-test. ****, $p < 0.0001$; ***, $p < 0.001$; *ns*, not significant.

(D) U2OS cells expressing mt-Keima were transduced the indicated dual sgRNAs. On day 8 post-transduction, cells were treated with Baf-A1 (100nM) or BTZ (100nM) 18hr prior to flow cytometry. Bar graphs represent mean +/- SEM from 3 independent experiments. Statistical analysis was performed using two-way ANOVA with Tukey's post-test. *, $p < 0.05$.

(E) MDA-MB-231 cells expressing mt-Keima were transduced with either a non-targeting sgRNA (sgCtrl) or sgEMC3. On day 8 post-transduction, cells were incubated with vehicle (DMSO) or Baf-A1 (100nM) for 18hr prior to flow cytometry. (n > 10,000 cells)

(F) MDA-MB-231 cells expressing mt-Keima were transduced the indicated dual sgRNAs. On day 8 post-transduction, cells were treated with Baf-A1 (100nM) for 18h or subjected to hypoxic conditions for 18h prior to flow cytometry. (n > 10,000 cells)

Appendix Figure S1

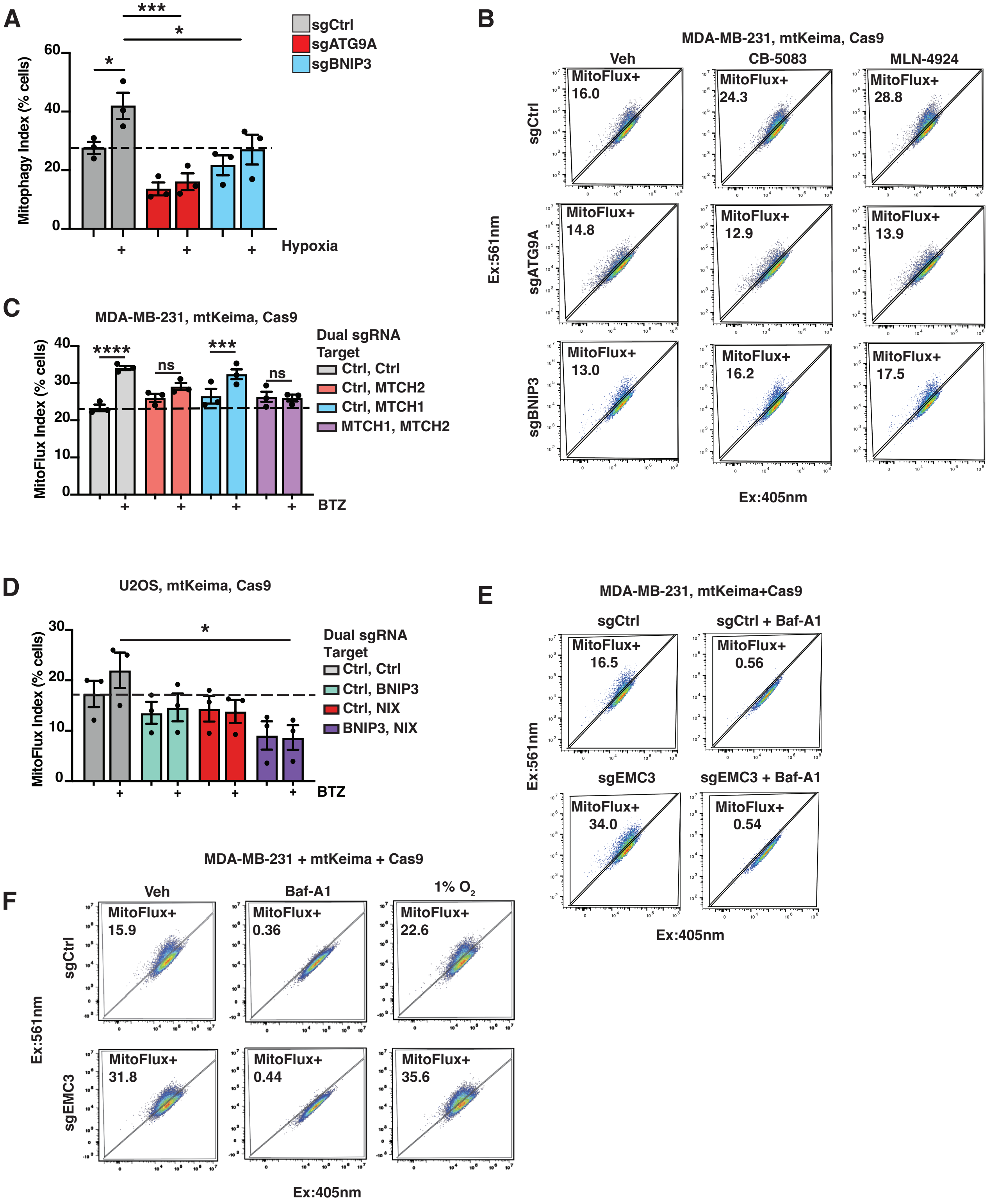

Supplement: Supplementary file 1 — Appendix [file 44318_2023_6_MOESM1_ESM.pdf]
