# Supplementary material for: The ER membrane protein complex restricts mitophagy by controlling BNIP3 turnover
Source: EMBO J. 2023 Dec 15;43(1):32–60. doi: 10.1038/s44318-023-00006-z (PMC10883272; doi:10.1038/s44318-023-00006-z)
Supplement: Supplementary file 4 — Table EV2 [file 44318_2023_6_MOESM4_ESM.docx]

**Table EV2: Source Data Identifiers**

| **Corresponding Figure** | **Description** | **File Type** | **DOI** |
| --- | --- | --- | --- |
| Figure 2B | CRISPR Screen Input | Fastq | 10.17632/68shkvyzrr.2 |
| Figure 2B | D8_BR: Sorted cells day 8 post-transduction, bottom right population (effectors) | Fastq | 10.17632/msypt5jm5g.2 |
| Figure 2B | D8_TL: Sorted cells day 8 post-transduction, top left population (suppressors) | Fastq | 10.17632/24zgw9789p.2 |
| Figure 2B | D11_TL: Sorted cells day 11 post-transduction, top left population (suppressors) | Fastq | 10.17632/5s48s2wznb.2 |
| Figure 2B | D11_BR: Sorted cells day 11 post-transduction, bottom right population (effectors) | Fastq | 10.17632/3kbd99k7c2.2 |
| Figure 2B | D12_TL: Sorted cells day 12 post-transduction, top left population (suppressors) | Fastq | 10.17632/gbb323nb9t.2 |
| Figure 2B | D12_BR: Sorted cells day 12 post-transduction, bottom right population (effectors) | Fastq | 10.17632/r2z86p7b7x.2 |
| Figure 6A | Raw Flow cytometry (fsc) | FCS | 10.17632/stzrvk8wyb.2 |
| Figure 7B | Raw Flow cytometry (fsc) | FCS | 10.17632/stzrvk8wyb.2 |
| Figure 1D | Representative microscopy images | Nd2 | 10.17632/7pgkgd6bz5.2 |
| Figure 1F | Representative microscopy images | Nd2 | 10.17632/7pgkgd6bz5.2 |
| Figure 3B | Representative microscopy images | Nd2 | 10.17632/z32xynk7jt.2 |
| Figure 3C | Representative microscopy images | Nd2 | 10.17632/rsgpscdmrz.2 |
| Figure 4B | Representative microscopy images | Nd2 | 10.17632/cc2th566m7.2 |
| Figure 5E | Representative microscopy images | Nd2 | 10.17632/nw4xxkt9p5.2 |
