# Supplementary figures and images for: The ER membrane protein complex restricts mitophagy by controlling BNIP3 turnover
Source: EMBO J. 2023 Dec 15;43(1):32–60. doi: 10.1038/s44318-023-00006-z (PMC10883272; doi:10.1038/s44318-023-00006-z)

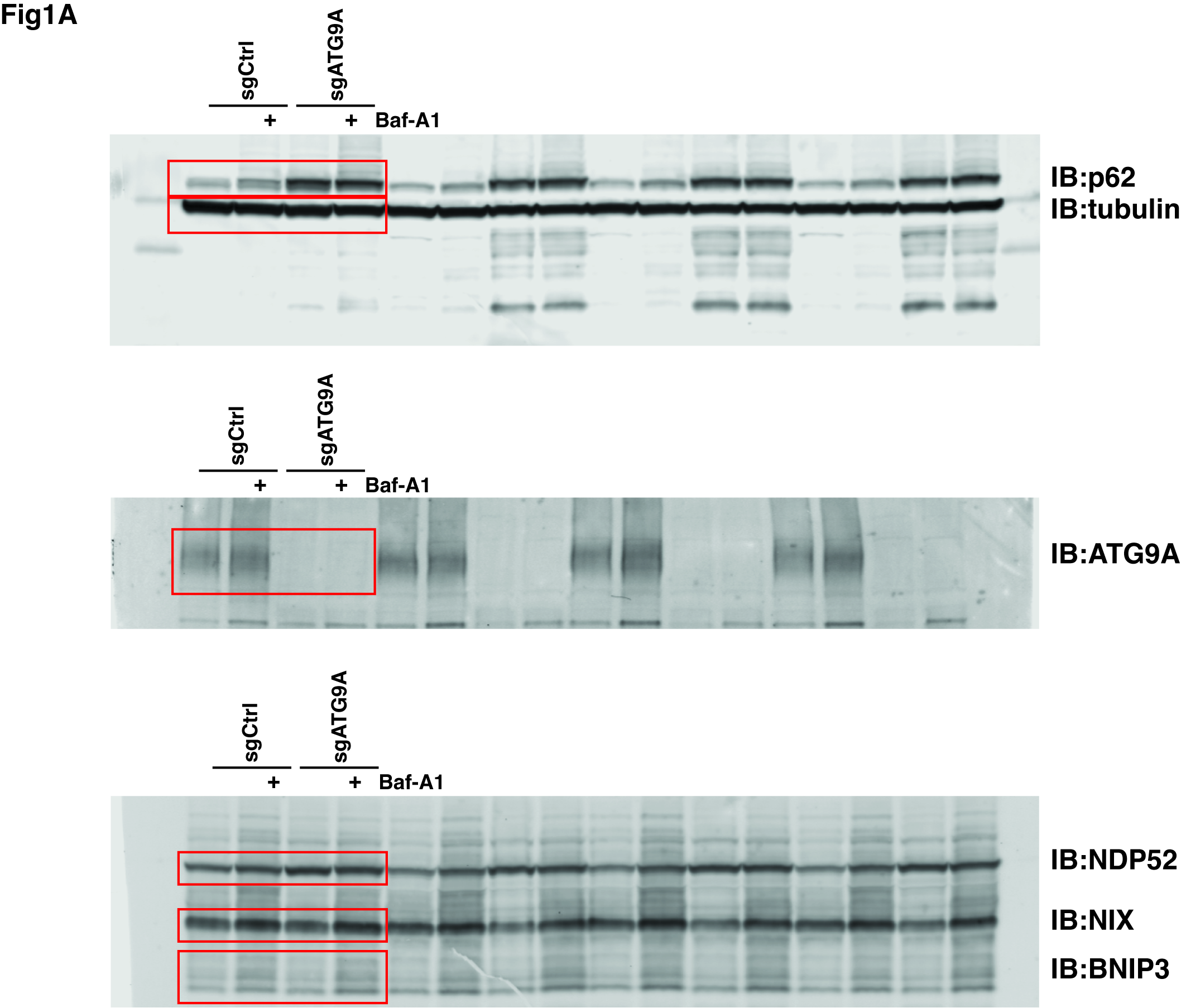

Supplement: Supplementary file 6 — Source Data Fig. 1 [file 44318_2023_6_MOESM6_ESM.zip › Fig1A.tif]

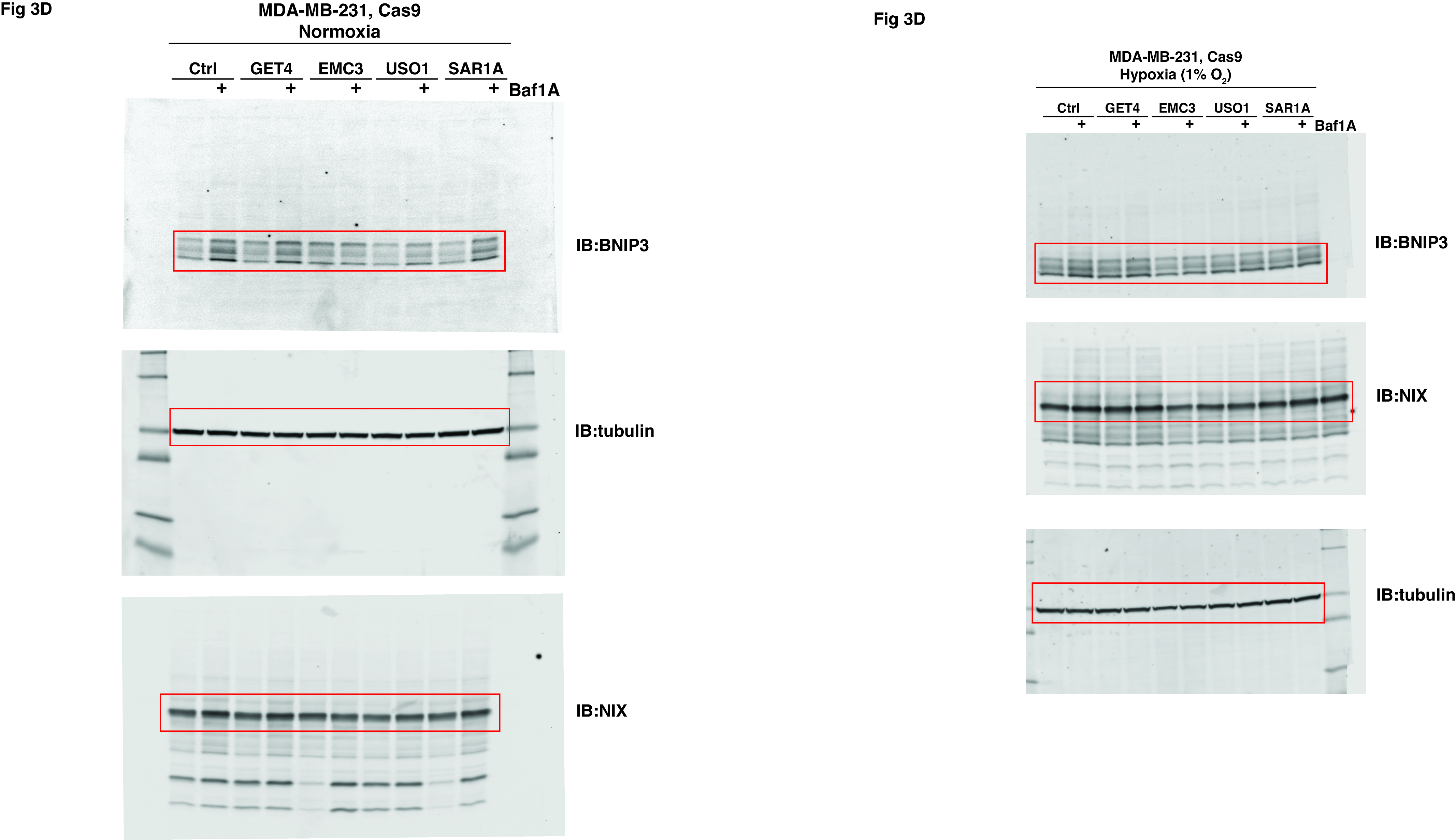

Supplement: Supplementary file 7 — Source Data Fig. 3 [file 44318_2023_6_MOESM7_ESM.zip › Fig3D.tif]

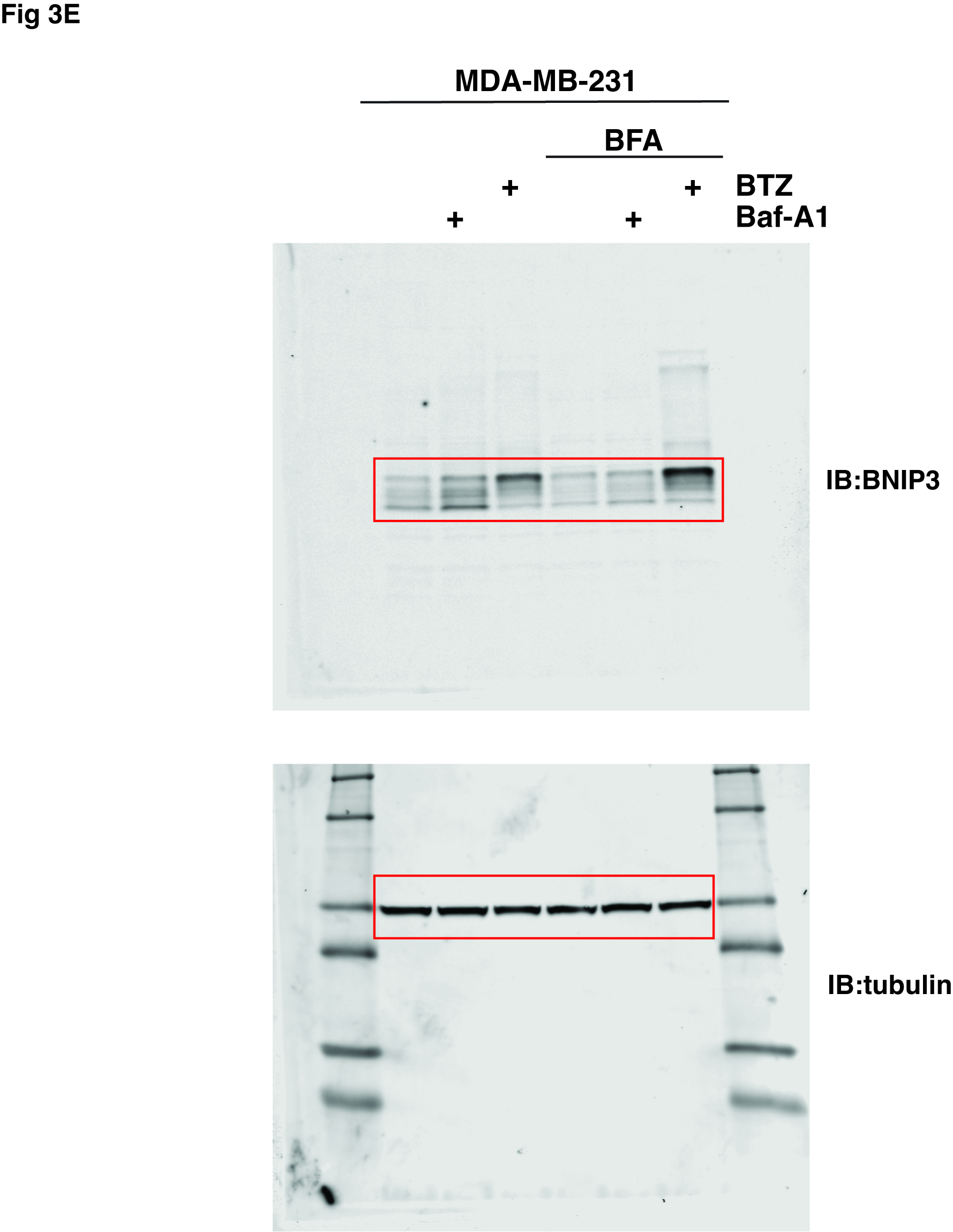

Supplement: Supplementary file 7 — Source Data Fig. 3 [file 44318_2023_6_MOESM7_ESM.zip › Fig3E.tif]

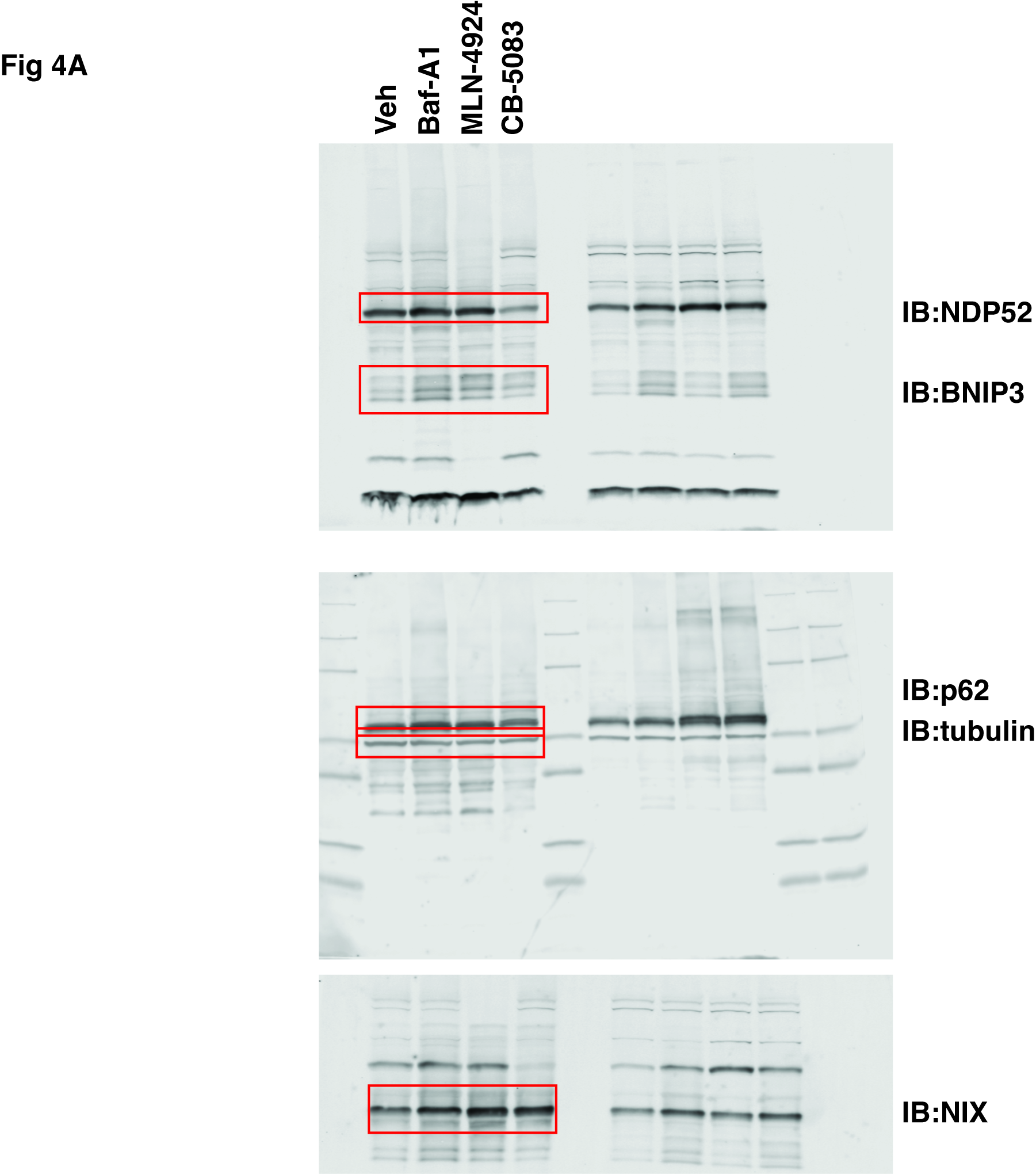

Supplement: Supplementary file 8 — Source Data Fig. 4 [file 44318_2023_6_MOESM8_ESM.zip › Fig4A.tif]

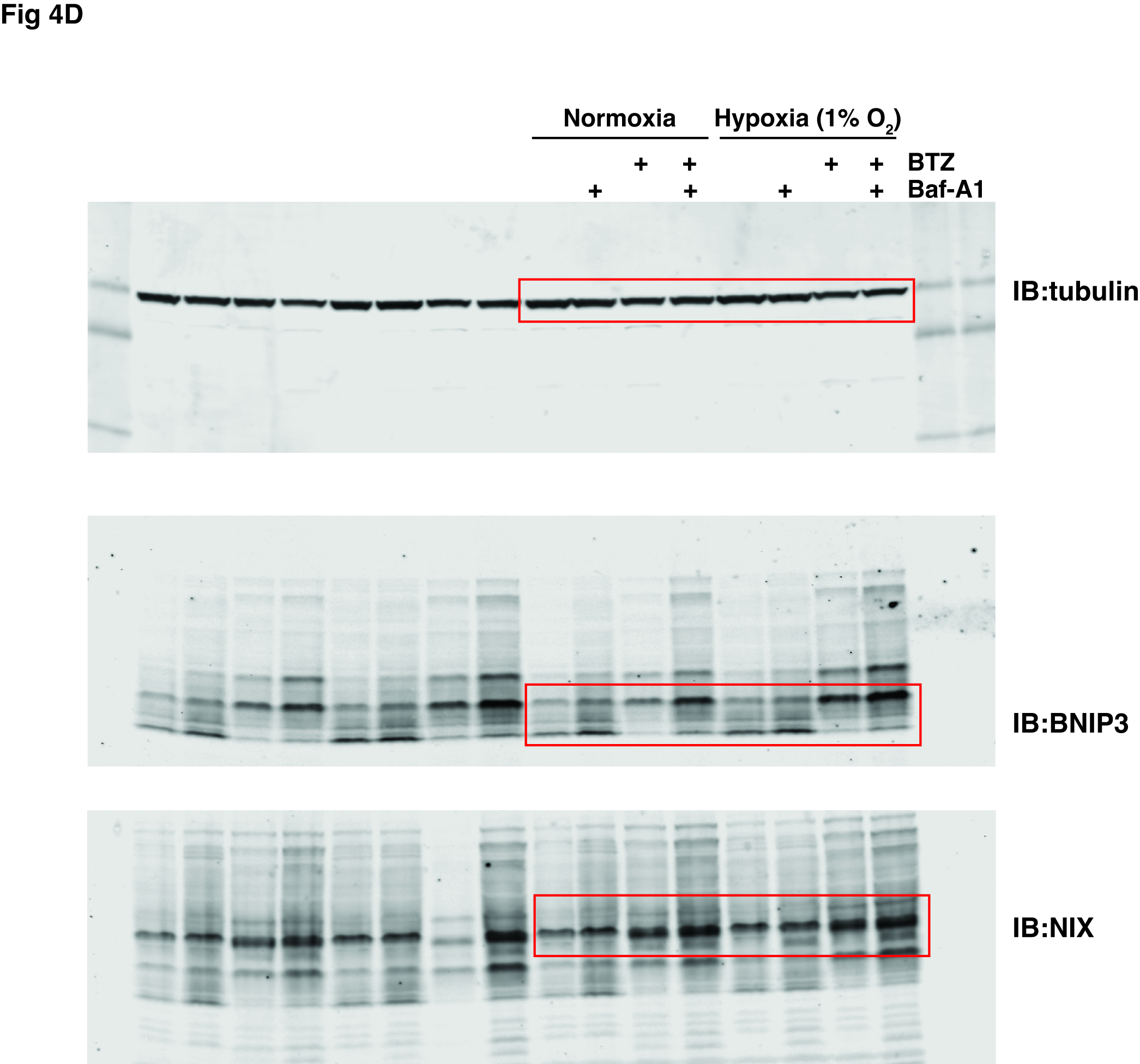

Supplement: Supplementary file 8 — Source Data Fig. 4 [file 44318_2023_6_MOESM8_ESM.zip › Fig4D.tif]

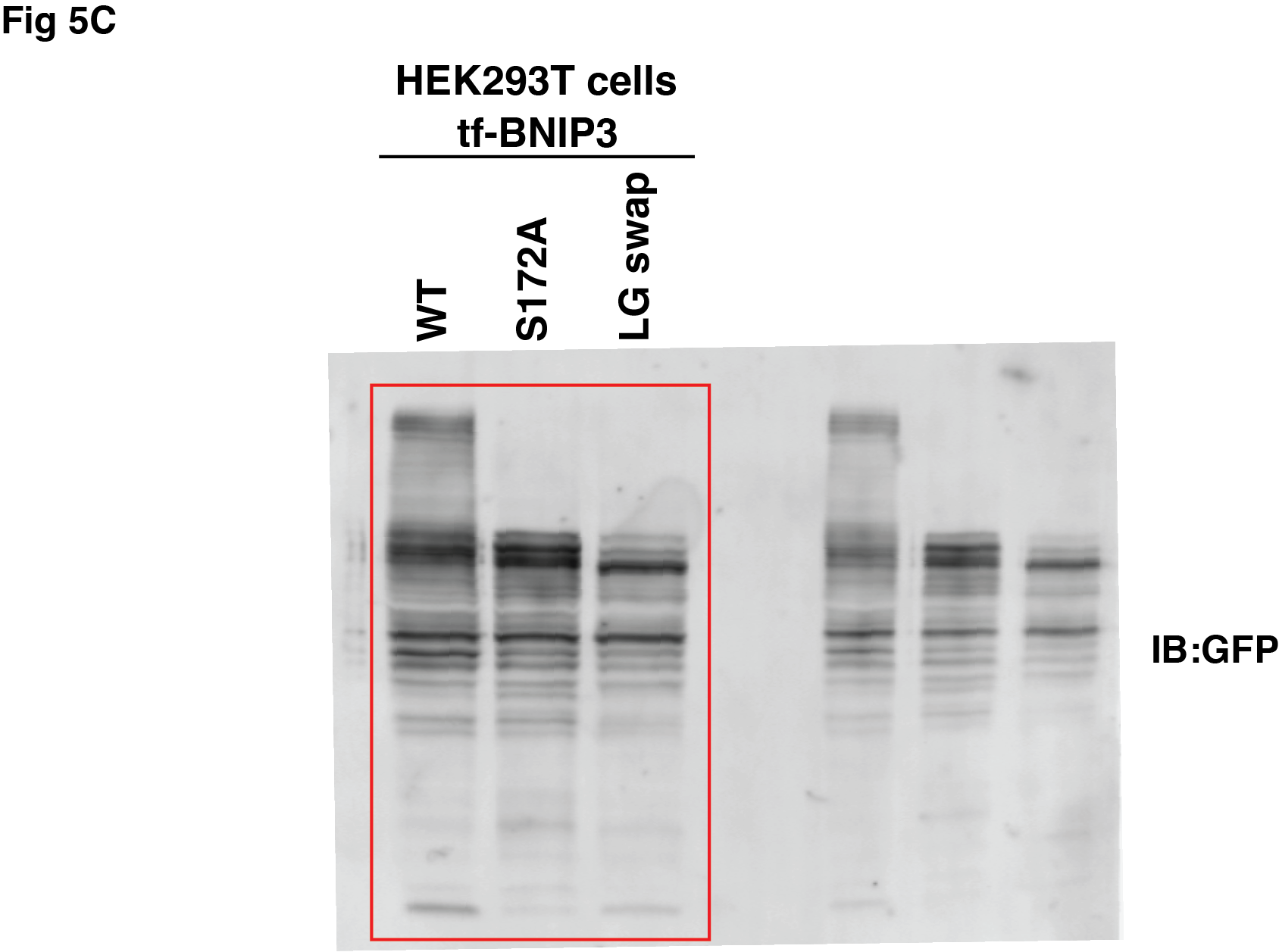

Supplement: Supplementary file 9 — Source Data Fig. 5 [file 44318_2023_6_MOESM9_ESM.zip › Fig5C.png]

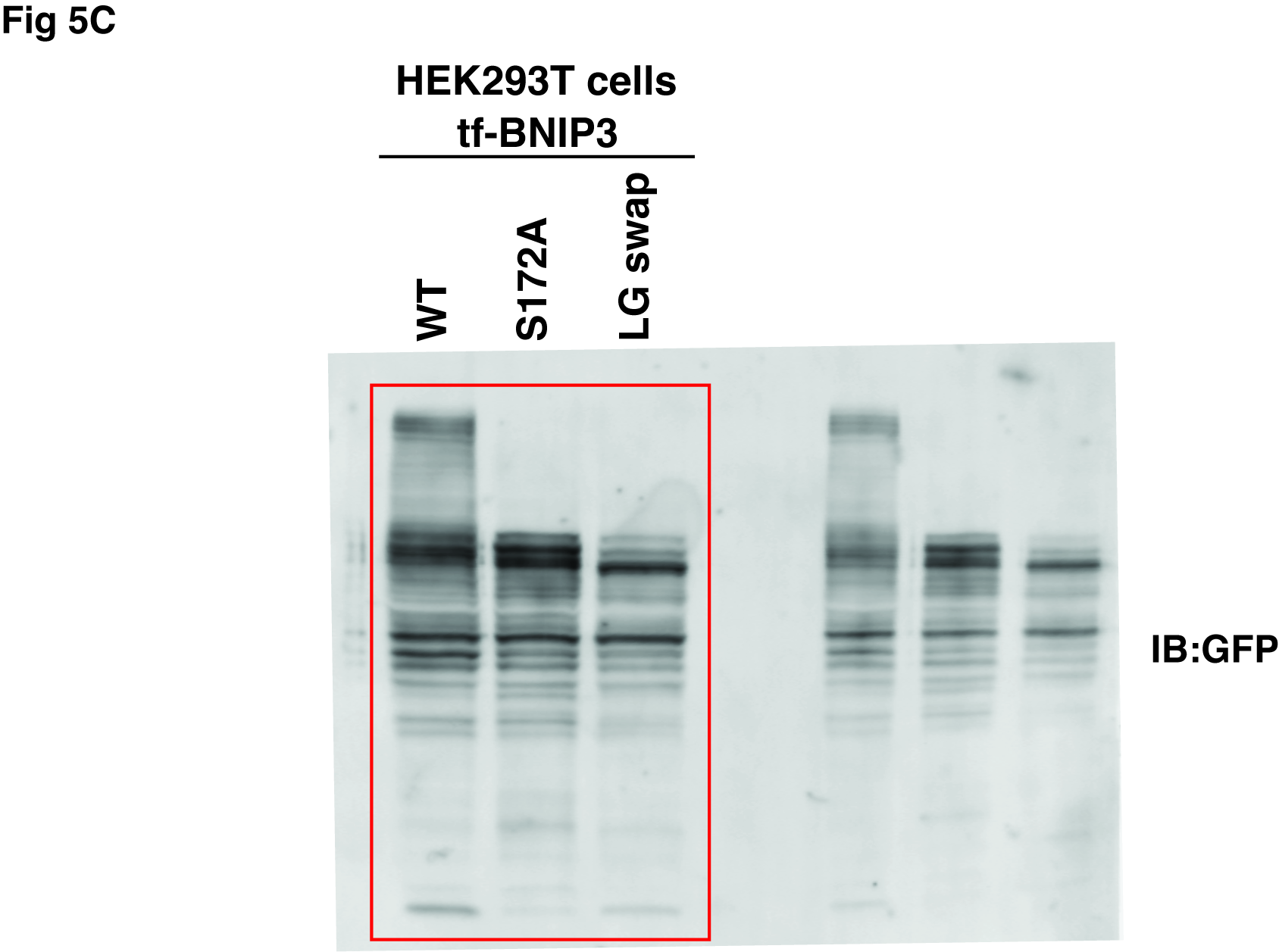

Supplement: Supplementary file 9 — Source Data Fig. 5 [file 44318_2023_6_MOESM9_ESM.zip › Fig5C.tif]
