# Supplementary material for: The ER membrane protein complex restricts mitophagy by controlling BNIP3 turnover
Source: EMBO J. 2023 Dec 15;43(1):32–60. doi: 10.1038/s44318-023-00006-z (PMC10883272; doi:10.1038/s44318-023-00006-z)
Supplement: Supplementary file 12 — Expanded View Figures [file 44318_2023_6_MOESM12_ESM.pdf]

Expanded View Figures

Figure EV1. Related to Fig. 1.

(**A**) MDA-MB-231 cells were transduced with V5-BNIP3 variants and lysed 48 h post transduction. The V5 epitope was immunoprecipitated from extracts and treated with buffer alone (lane 1), lambda phosphatase (PP, lane 2), or lambda phosphatase with phosphatase inhibitor cocktail (PIC, lane 3). (**B**) Immunoblotting of MDA-MB-231-derived extracts from wild-type (WT) and *ATG9*^{KO} clonal knockout cells. Where indicated, cells were treated with Baf-A1 (100 nM) for 18 h. (**C–E**) Immunoblotting of MDA-MB-231, K562, U2OS, MDA-MB-453-derived extracts from cells expressing Cas9 and the indicated sgRNA. Cells were subjected to normoxia or hypoxia and/or Baf-A1 treatment (100 nM) for 18 h where indicated. (**F**) Immunoblotting of extracts derived from parental HEK293T and clonal *ATG9*^{KO} knockout cells. Where indicated, cells were treated with Baf-A1 (100 nM) for 18 h. (**G**) Violin plots of MDA-MB-231 cells expressing either the tf-NDP52 or tf-BNIP3 reporter. Cells were treated with DMSO or Baf-A1 (100nM) or PIK-III (10 μM) for 18 h before being analyzed by flow cytometry for red:green ratio. Median values for each sample are identified by a black line within each violin. The red dotted line across all samples corresponds to red:green ratio of maximally inhibited conditions (Baf-A1) (n >10,000 cells). (**H**) Violin plots of MDA-MB-231 cells expressing tf-BNIP3 transduced with either a control small hairpin RNA (shCtrl) or an shRNA targeting Rab7 (shRab7). Cells were analyzed for red:green ratio 8 days post transduction. Red dotted line (=1) corresponds to the theoretical maximum inhibition of red:green ratio. (n >10,000 cells). (**I**) Quantification of Pearson's correlation coefficients from cells in Fig. 1D. Correlation of RFP to mitoBFP (reflective of mitochondrial localization) was calculated using Coloc2. Bar graphs represent mean +/− SEM. Each data point represents a single cell. n = 15 cells. Statistical analysis was performed using an unpaired Student's t test. *P<0.05.

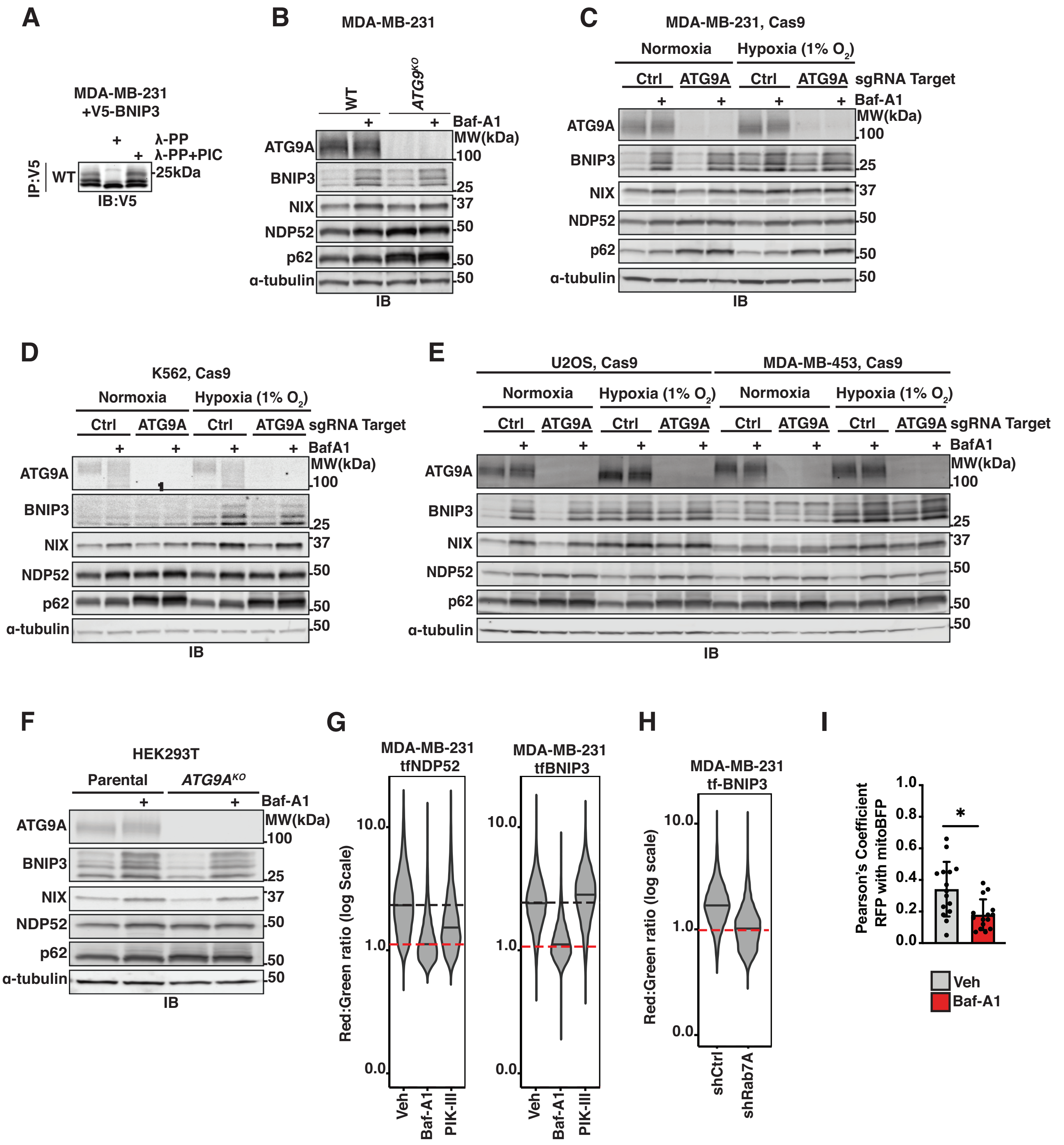
A
MDA-MB-231 +V5-BNIP3
λ-PP
λ-PP+PIC
IP:V5
WT
25kDa
IB:V5
B
MDA-MB-231
WT
ATG9KO
Baf-A1
ATG9A
BNIP3
NIX
NDP52
p62
α-tubulin
MW(kDa)
IB
C
MDA-MB-231, Cas9
Normoxia
Hypoxia (1% O2)
Ctrl
ATG9A
sgRNA Target
D
K562, Cas9
BafA1
E
U2OS, Cas9
MDA-MB-453, Cas9
F
HEK293T
Parental
ATG9AKO
G
MDA-MB-231 tfNDP52
MDA-MB-231 tfBNIP3
Red:Green ratio (log Scale)
Veh
Baf-A1
PIK-III
H
MDA-MB-231 tf-BNIP3
Red:Green ratio (log scale)
shCtrl
shRab7A
I
Pearson's Coefficient RFP with mitoBFP
Veh
Baf-A1

 © The Author(s)

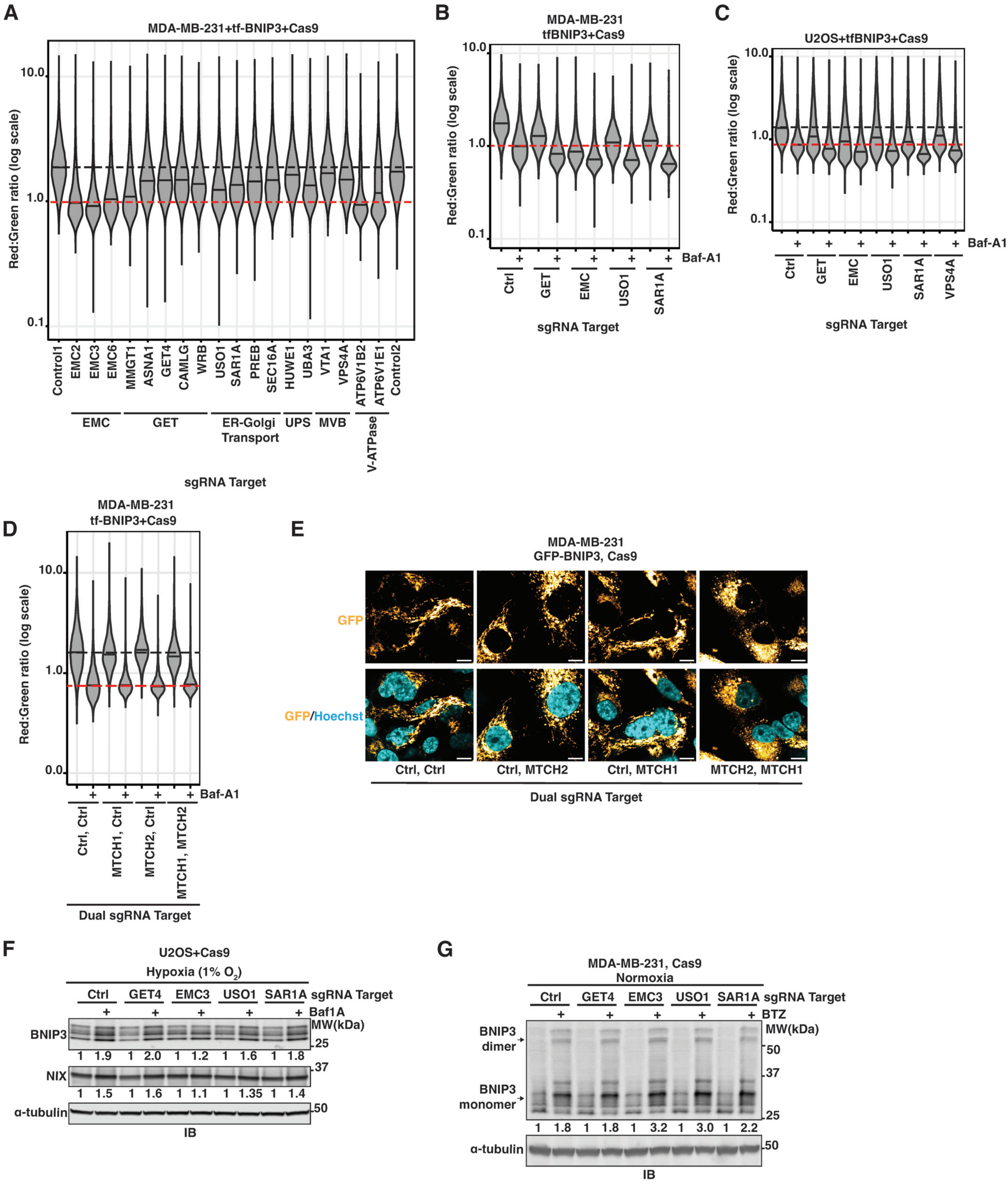
A
MDA-MB-231+tf-BNIP3+Cas9
Red:Green ratio (log scale)
10.0
1.0
0.1
Control1 EMC2 EMC3 EMC6 MMGT1 ASNA1 GET4 CAMLG WRB USO1 SAR1A PREB SEC16A HUWE1 UBA3 VTA1 VPS4A ATP6V1B2 ATP6V1E1 Control2
EMC
GET
ER-Golgi Transport
UPS
MVB
V-ATPase
sgRNA Target
B
MDA-MB-231
tfBNIP3+Cas9
Red:Green ratio (log scale)
10.0
1.0
0.1
Baf-A1
Ctrl GET EMC USO1 SAR1A
sgRNA Target
C
U2OS+tfBNIP3+Cas9
Red:Green ratio (log scale)
10.0
1.0
0.1
Baf-A1
Ctrl GET EMC USO1 SAR1A VPS4A
sgRNA Target
D
MDA-MB-231
tf-BNIP3+Cas9
Red:Green ratio (log scale)
10.0
1.0
0.0
Baf-A1
Ctrl, Ctrl
MTCH1, Ctrl
MTCH2, Ctrl
MTCH1, MTCH2
Dual sgRNA Target
E
MDA-MB-231
GFP-BNIP3, Cas9
GFP
GFP/Hoechst
Ctrl, Ctrl
Ctrl, MTCH2
Ctrl, MTCH1
MTCH2, MTCH1
Dual sgRNA Target
F
U2OS+Cas9
Hypoxia (1% O2)
Ctrl GET4 EMC3 USO1 SAR1A sgRNA Target
Baf1A
MW(kDa)
BNIP3
25
1 1.9 1 2.0 1 1.2 1 1.6 1 1.8
37
NIX
1 1.5 1 1.6 1 1.1 1 1.35 1 1.4
α-tubulin
50
IB
G
MDA-MB-231, Cas9
Normoxia
Ctrl GET4 EMC3 USO1 SAR1A sgRNA Target
BTZ
MW(kDa)
BNIP3 dimer
50
37
BNIP3 monomer
25
1 1.8 1 1.8 1 3.2 1 3.0 1 2.2
α-tubulin
50
IB

Figure EV2. Related to Fig. 3.

(**A**) MDA-MB-231 cells expressing tf-BNIP3 and Cas9 were transduced with the indicated sgRNAs. Red:green ratio was analyzed by flow cytometry on day 8 post transduction. Median values for a nontargeting control (sgControl1) are identified by a dashed black line. The red dotted line across all samples corresponds to a red:green ratio of 1, the theoretical minimum (n >10,000 cells). (**B**) MDA-MB-231 cells expressing tf-BNIP3 and Cas9 were transduced with the indicated sgRNAs. Red:green ratio was analyzed by flow cytometry on day 8 post transduction. The red dotted line across all samples corresponds to red:green ratio of Baf-A1-treated control (Ctrl) cells (n >10,000 cells). (**C**) U2OS cells expressing tf-BNIP3 and Cas9 were transduced with the indicated sgRNAs. Red:green ratio was analyzed by flow cytometry on day 8 post transduction. The red dotted line across all samples corresponds to red:green ratio of Baf-A1-treated control (Ctrl) cells (n >10,000 cells). (**D**) MDA-MB-231 cells expressing tf-BNIP3 and Cas9 were transduced with the indicated dual sgRNAs. Red:green ratio was analyzed by flow cytometry on day 8 post transduction. The red dotted line across all samples corresponds to red:green ratio of Baf-A1-treated control (Ctrl) cells (n > 10,000 cells). (**E**) Representative confocal micrographs of MDA-MB-231 cells expressing GFP-BNIP3 and Cas9. Cells were transduced with indicated sgRNAs, propagated for 8 days and fixed for image aquisition. Hoechst stain was used for nuclear staining. Scale bar is 10 µm. (**F**) Immunoblotting of U2OS-derived extracts expressing Cas9 that were transduced with the indicated sgRNAs. On day 8 post transduction, cells were treated with Baf-A1 (100 nM) and subjected to hypoxia for 18 h prior to lysis. (**G**) Immunoblotting of MDA-MB-231-derived extracts expressing Cas9 that were transduced with the indicated sgRNAs. Cells were treated with Bortezomib (100 nM) for 18 h on day 8 post transduction, prior to lysis.

 © The Author(s)

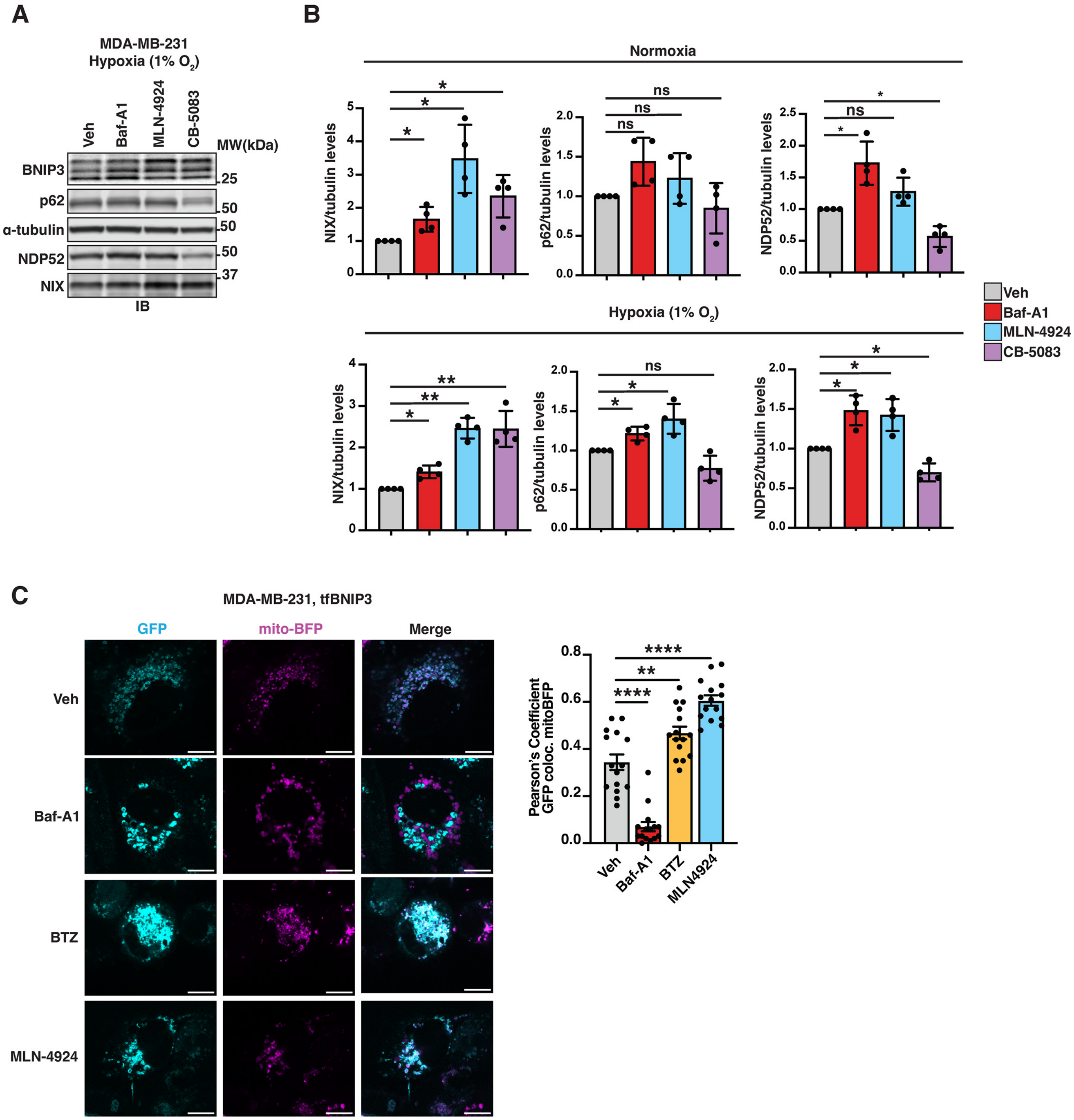

Figure EV3. Related to Fig. 4.

(**A**) Representative image of one biological replicate quantified in Fig. 4A. Immunoblotting (IB) of MDA-MB-231-derived extracts from cells treated with vehicle (DMSO), Baf-A1 (100 nM), MLN-4924 (1 μM), CB-5083 (1 μM) and subjected to hypoxia for 18 h. (**B**) Quantification of protein accumulation from Figs. 4D and EV3A. Bar graphs represent mean +/− SEM from four independent experiments. All protein levels were normalized to α-tubulin. Statistical analysis was performed using a one-sample *t* test to the normalized control. ***P*< 0.01; **P*<0.05; ns not significant. (**C**) Representative confocal micrographs of tf-BNIP3-expressing cells treated with Baf-A1 (100 nM), MLN-4924 (1 μM), or Bortezomib (100 nM) for 18 h. Pearson's correlation coefficient between GFP and mitoBFP (reflective of mitochondrial localization) was calculated using Coloc2. Bar graphs represent mean +/− SEM. Each data point represents a single cell. Statistical analysis was performed using an unpaired t test. Scale bar: 10 μm; *n* = 15 cells; *****P*<0.0001, ***P*<0.01.

© The Author(s)

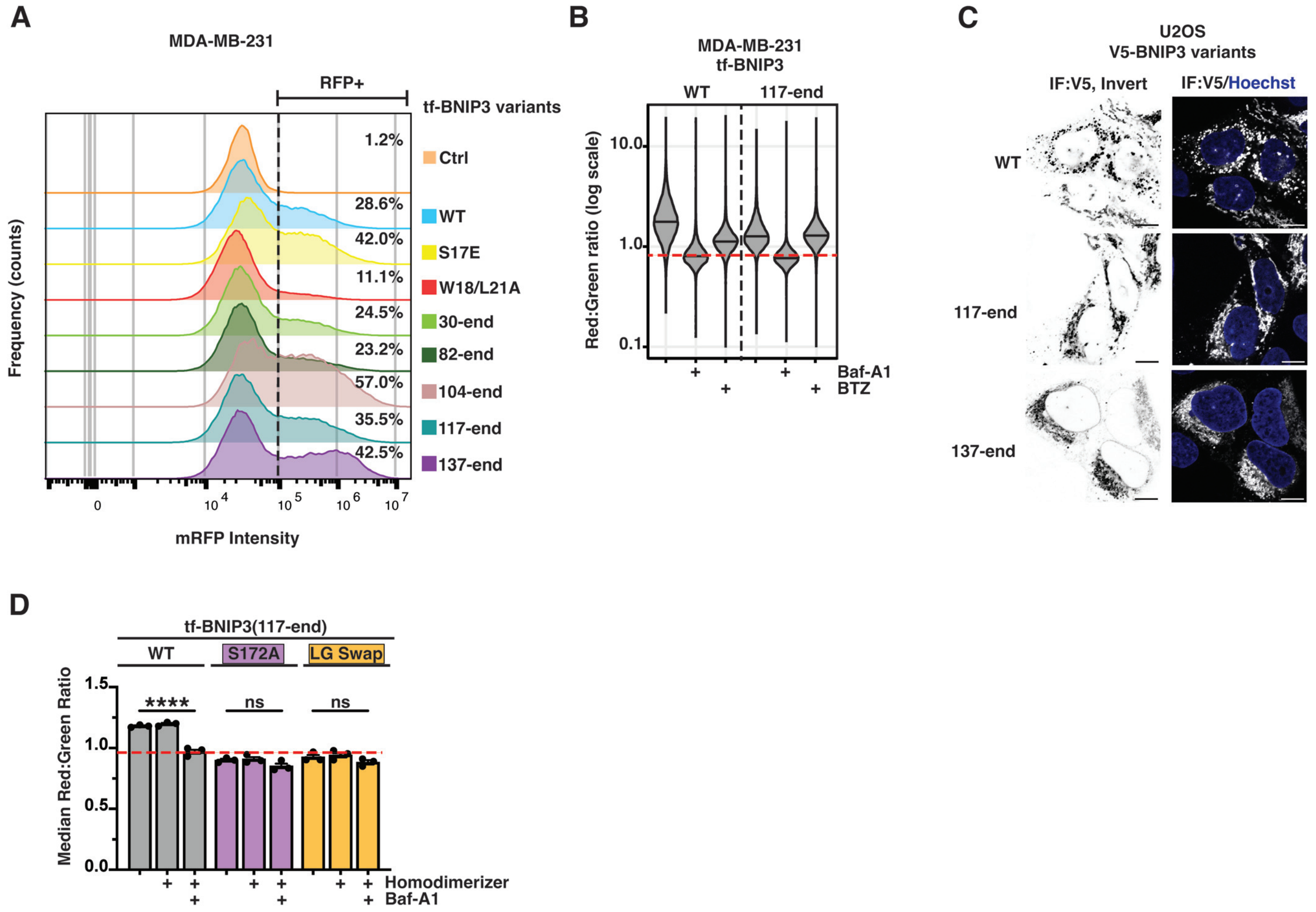

Figure EV4. Related to Fig. 5.

(**A**) Histograms for expression of tf-BNIP3 variants. MDA-MB-231 cells were transduced with the indicated tf-BNIP3 variants and analyzed by flow cytometry 48 h post transduction. Threshold for mRFP+ cells was determined by a non-transduced control (Ctrl). The percentage of RFP+ cells are indicated for each sample. (*n* > 10,000 cells). (**B**) MDA-MB-231 cells were transduced with the indicated tf-BNIP3 variants. Red:green ratio was analyzed by flow cytometry 48 h post transduction. Cells were treated with vehicle (DMSO), Baf-A1 (100 nM), or BTZ (100 nM) for 18 h prior to performing flow cytometry. The red dotted line across each sample group corresponds to the maximum inhibition red:green ratio of the wild-type (WT) Baf-A1-treated sample (*n* > 10,000 cells). (**C**) Representative confocal micrographs of U2OS cells transduced with V5-BNIP3 variants. 48 h post transduction, cells were fixed and immunostained for the V5 epitope. Hoechst stain was used for nuclear staining. Scale bar is 10 µm. (**D**) MDA-MB-231 cells were transduced with the indicated tf-BNIP3$^{117\text{-end}}$ variants. Red:green ratio was analyzed by flow cytometry 48h post transduction. Cells were treated with Baf-A1 (100 nM) and/or B/B homodimerizer (0.5 µM) for 6 h prior to performing flow cytometry. Bar graphs represent mean +/− SEM from three independent experiments. The red dotted line across all samples corresponds to cells inhibited with Baf-A1 (*n* > 10,000 cells). Statistical analysis was performed using a two-way ANOVA with Dunnett's test. ****P<0.0001.

 © The Author(s)

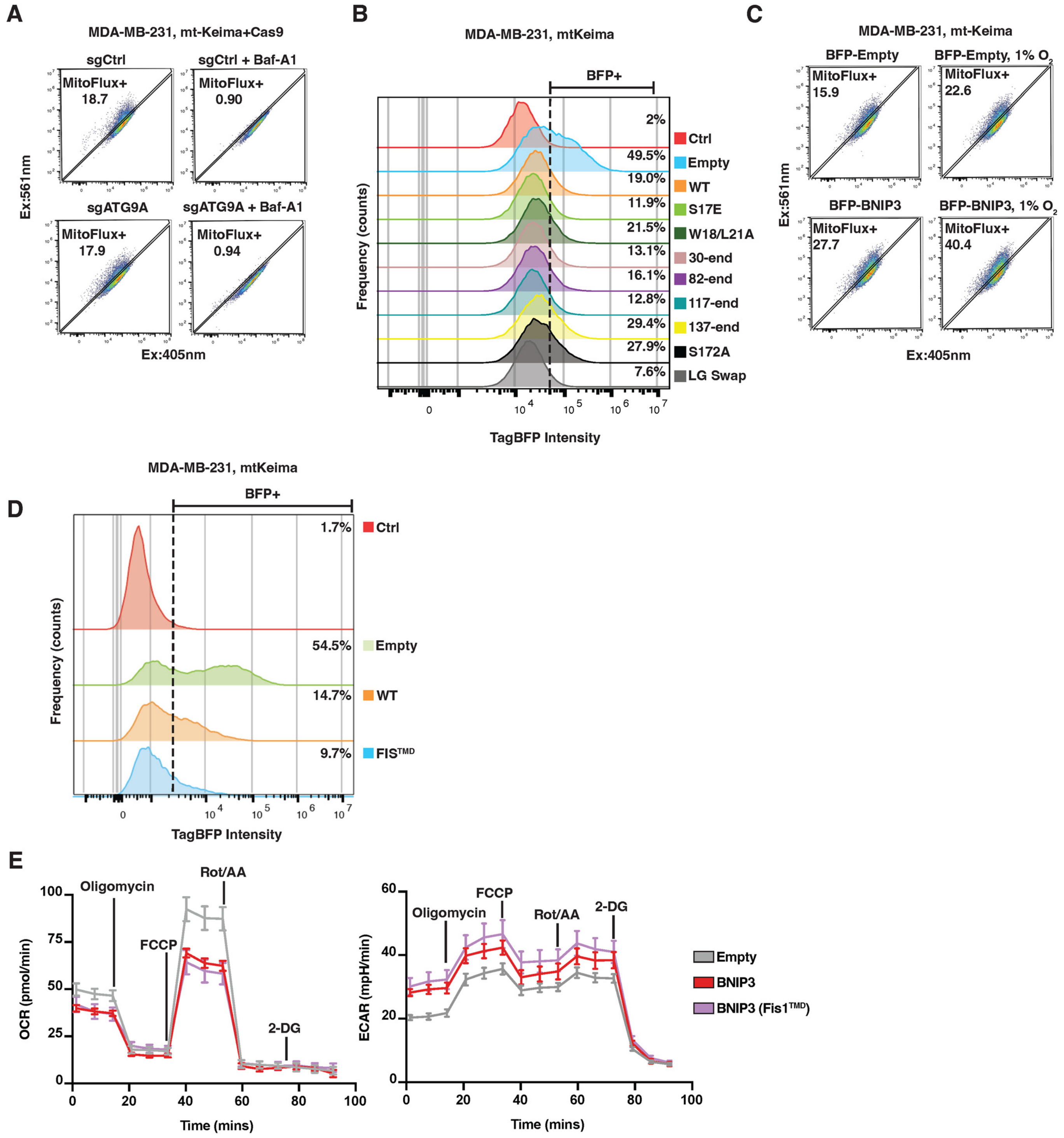

Figure EV5. Related to Fig. 6.

(**A**) MDA-MB-231 cells expressing mt-Keima were transduced with either a nontargeting sgRNA (sgCtrl) or sgATG9A. On day 8 post transduction, cells were incubated with vehicle (DMSO) or Baf-A1 (100 nM) for 18 h and assessed by flow cytometry. (n > 10,000 cells). (**B**) Histograms for expression of BFP-BNIP3 variants. MDA-MB-231 mt-Keima cells were transduced with the indicated TagBFP-BNIP3 variants and analyzed by flow cytometry 48 h post transduction. Threshold for TagBFP+ cells was determined by a non-transduced control (Ctrl). The percentage of TagBFP+ cells are indicated for each sample. (n > 10,000 cells). (**C**) MDA-MB-231 cells expressing mt-Keima were transduced with BFP-BNIP3. At 24 h post transduction, cells were incubated in normoxic or hypoxic conditions for 18 h and assessed by flow cytometry. (n > 10,000 cells). (**D**) Histograms for expression of BFP-BNIP3 variants. MDA-MB-231 mt-Keima cells were transduced with the indicated TagBFP-BNIP3 variants and analyzed by flow cytometry 48 h post transduction. Threshold for TagBFP+ cells was determined by a non-transduced control (Ctrl). Percentage of TagBFP+ cells are indicated for each sample. (n > 10,000 cells). (**E**) MDA-MB-231 cells were transduced with indicated the BFP-BNIP3 variants and analyzed for oxygen consumption rate (OCR) and extracellular acidification rate (ECAR) 48 h post transduction. Values were normalized by BCA protein assay. Graphs represent the mean +/− SEM from five technical replicates.

© The Author(s)
